# Percolation on Networks with Conditional Dependence Group

**DOI:** 10.1371/journal.pone.0126674

**Published:** 2015-05-15

**Authors:** Hui Wang, Ming Li, Lin Deng, Bing-Hong Wang

**Affiliations:** 1 School of Computer and Information/Hefei University of Technology, Hefei, Anhui Province, 230009, P.R. China; 2 Department of Modern Physics/University of Science and Technology of China, Hefei, Anhui Province, 230026, P.R. China; 3 Information Construction and Development Center/Hefei University of Technology, Hefei, Anhui Province, 230009, P.R. China; 4 Center of Information Support and Assurance Technology, Anhui University, Hefei, Anhui Province, 230601, P.R. China; Beihang University, CHINA

## Abstract

Recently, the dependence group has been proposed to study the robustness of networks with interdependent nodes. A dependence group means that a failed node in the group can lead to the failures of the whole group. Considering the situation of real networks that one failed node may not always break the functionality of a dependence group, we study a cascading failure model that a dependence group fails only when more than a fraction *β* of nodes of the group fail. We find that the network becomes more robust with the increasing of the parameter *β*. However, the type of percolation transition is always first order unless the model reduces to the classical network percolation model, which is independent of the degree distribution of the network. Furthermore, we find that a larger dependence group size does not always make the networks more fragile. We also present exact solutions to the size of the giant component and the critical point, which are in agreement with the simulations well.

## Introduction

In the last decades, complex networks have attracted increasing attention [1, 2], since many realistic systems can be described by networks, such as the Internet, metabolic system, food web and traffic system [3]. One of the important topics in complex networks is the robustness of the networked system, i.e., the ability of a network to resist change without adapting its initial stable configuration.

In theory, network percolation model is usually used to study the robustness of networks [4–6]. In simple terms, the network percolation model describes the behavior of connected clusters of a network after a fraction 1 − *p* of nodes have been removed. This model usually exhibits a second order phase transition, that is the size of the giant component continuously decreases to zero while *p* decreases to the critical point *p*_*c*_. In addition, the critical point *p*_*c*_ is usually used to evaluate the robustness of the networks. The smaller the critical point *p*_*c*_ is, the more robust the network is. For some specific cases, such as random networks, the critical point can be solved exactly [7, 8].

Recently, to study the effects of the dependence between nodes on the robustness of network, the concept of dependence group (link) has been proposed [9, 10]. The dependence group means that if one of the nodes in a group fails, the group will fail totally, i.e., all the other nodes of the group fail. For example, in a financial network, the trading and sale connections between companies can be understood as the connectivity links of the network. Besides, the companies in the same industrial chain could form a dependence group. If one company fails, the other companies in the same industrial chain could also fail due to the rupture of the industrial chain.

Parshani *et al* find that the robustness of such networks is determined by the size *g* of the dependence group [10, 11]. A lager *g* could make such networks much more fragile than the networks without dependence group (*g* = 1), and the networks are more robust when the degrees are distributed more broadly. Furthermore, instead of the second order percolation transition, the networks with dependence group demonstrate a first order percolation transition for *g* > 1. In ref.[12], Bashan *et al* find that ER(Erdos-Rényi) and RR(random-regular) networks topologies differ greatly in their stability in the case of large dependence group sizes. In addition, when such networks are embedded into a two-dimensional space, they will be extremely fragile [13].

After that, Li *et al* also show that the overlapping of the connectivity and dependence links (*g* = 2) and the asymmetric dependence can make such networks more robust [14, 15]. In addition, the networks with dependence groups or links have also been studied in the form of interdependent networks and multilayer networks, which also shows the fragility of networks when nodes depend on each other [16, 17]. In these studies, some new phenomena have also been found, such as assortativity decreases the robustness of interdependent networks [18], percolation transitions are not always sharpened by making networks interdependent [19], simultaneous first and second order percolation transitions [20].

However, in reality, the failure of one node may not always lead to the failure of the dependence group. For example, in an industrial chain, two or more companies may play a similar role. Thus, if one of them fails, the other companies can also form a complete industrial chain. But if all these companies fail, the missing links will make it impossible for the other companies to form a complete industrial chain. As a result, all the other companies in this industrial chain will fail. We can find the similar phenomenon when we consider the rumor spreading on online social networks. Individuals on Facebook or Twitter always belong to some groups based on interest, work, or region. Someone in a group will listen to a rumor, only when a fraction of individuals in the same group listen to this rumor. Therefore, in reality, the dependence of nodes in a group could be conditional, only when a fraction of nodes fail, the whole group will fail.

**Fig 1:**
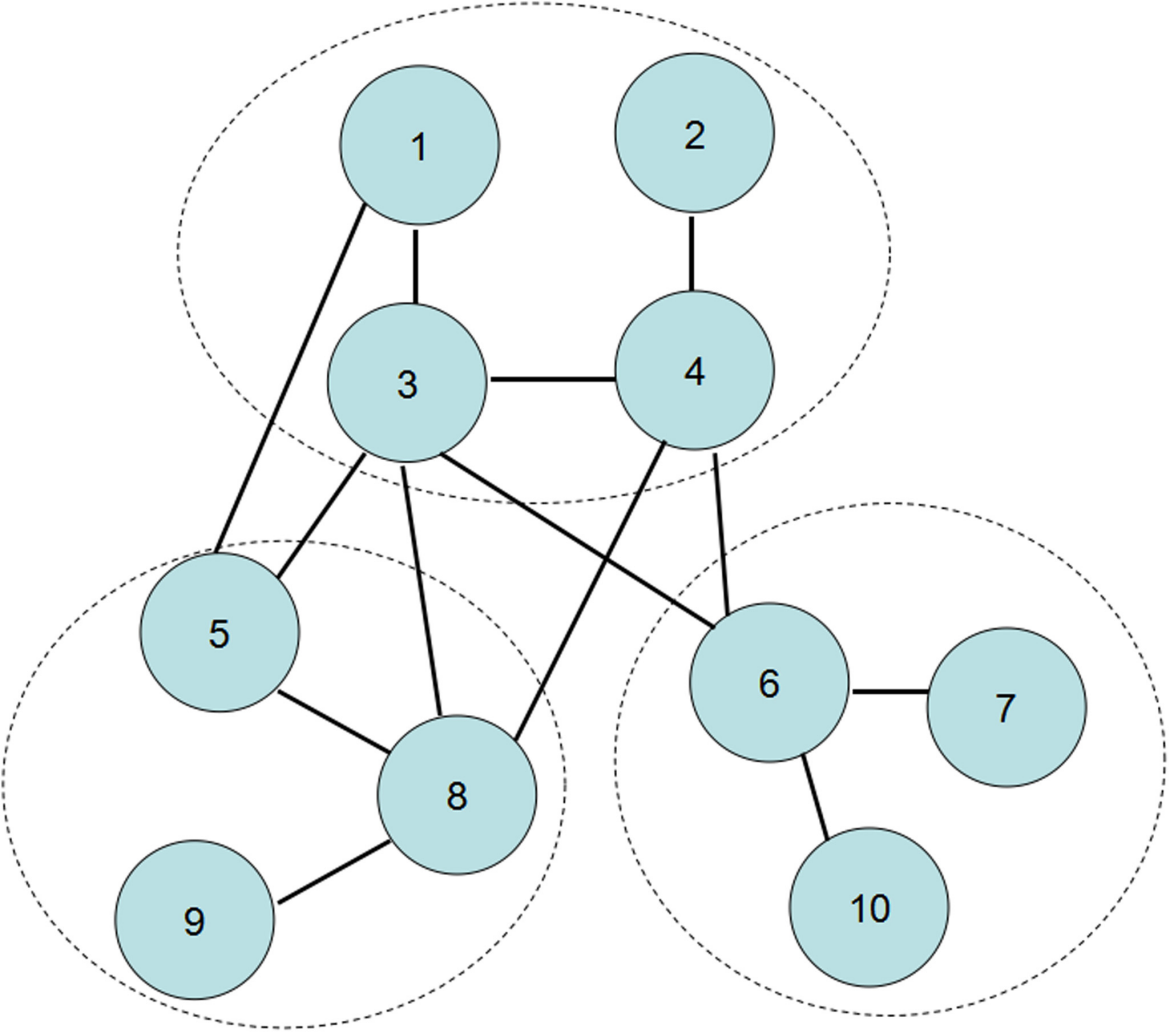
Networks with conditional dependence groups. The network is composed of nodes and connectivity links, and each node belongs to a dependence group (surrounded by dash lines). In each group, if more than a fraction *β* of nodes fail, the group will fail, i.e., all the nodes of this group fail. Take *β* = 0.5 as an example. In the group formed by nodes 5, 8 and 9, when node 9 fails, nodes 5 and 8 will still work since the fraction of failed nodes is less than *β*. However, if nodes 5 and 9 fail simultaneously, node 8 must also fail. This model can also be described by the load model used in ref.[15], this article does not cover details of this model.

In this paper, we will study the cascading dynamics of networks with this conditional dependence group. Following the model proposed by Parshani *et al* [10], we consider the network with a degree distribution *p*_*k*_ and each node belongs to a dependence group with size *g*. In our model, only when the fraction of the failed nodes of a group is larger than *β*, the group will fail, i.e., all nodes in the group fail. An illustration of this model is given in Fig 1. Obviously, our model is equivalent to the model proposed by Parshani *et al* when *β* → 0 [10], and reduces to the classical network percolation model when *β* → 1. In some sense, the parameter *β* represents the dependence intensity of the nodes in a group. A large *β* means the dependence of nodes is strong, one failed node could lead to the failure of the whole group. While the small *β* means the dependence of nodes is weak, one failed node does not affect the functionality of the group. Note that this model is different from the one proposed in ref.[15]. In their model, the failure of a group is determined by the total degrees of the failed nodes, and the nodes with larger degrees are more important for a group to keep its functionality. So the nodes in their model are unequal, the dependence is asymmetric. However, the dependence in our model is mutual, all the nodes in a group play the same role in keeping the group running.

The paper is organized as follows. In the next section, we will give the analytical results of our model using generating function techniques. And then, we perform computer simulations on ER networks to confirm the predictions of theoretical analysis. The discussion and conclusion are given in the last section.

## Analysis

The iterative process of cascading failures on the network with dependence groups begins by randomly removing a fraction 1 − *p* of nodes of the network as the initial failed nodes. The failed nodes will lead to two cascading failure processes:

1. Links that belong to the failed nodes will also fail, which could result in the other nodes disconnecting to the network. This process is called percolation step.
2. If the fraction of the failed nodes of a dependence group is larger than *β*, the group will fail, i.e., all the other nodes in this group fail. This process is called dependence step.

Once the cascade process begins, the two steps will occur alternately until there is no further splitting of nodes.

We solve this model by the method used in ref.[14, 21], which considers the final state after the cascades. In this paper, the probability that a randomly chosen node belongs to the giant component is denoted as *S*, which is the order parameter usually used in percolation theory. Furthermore, the parameter *R* gives the probability that a randomly chosen link connects to the giant component. Therefore, for the steady state, *S* satisfies

$$\begin{array}{c} S=p[1-G_{0}\left(1-R\right)]f\left(R\right). \end{array}$$ (1)

Here, *G*_0_(*x*) is the generating function of the degree distribution *G*_0_(*x*) = ∑_*k*_ *p*_*k*_ *x* ^*k*^. So it is easy to know that *p*[1−*G*_0_(1−*R*)] gives the probability that a randomly chosen node is not removed at the initial and belongs to the giant component. The function *f*(*R*) expresses the probability that the dependence group which the chosen node belongs to is functioning.

Then, we check that the conditions of a dependence group is functioning after the cascades, and give the expression of *f*(*R*). As the setting of the model, a functioning group can not have more than a fraction *β* of nodes that do not belong to the giant component. This yields

$$\begin{array}{c} f\left(R\right)=\sum_{i=0}^{\left\lfloor g\beta \right\rfloor }\left(\begin{array}{c} g-1\\ i \end{array}\right){\left\{1-p[1-G_{0}\left(1-R\right)]\right\}}^{i}{\left\{p[1-G_{0}\left(1-R\right)]\right\}}^{g-1-i}, \end{array}$$ (2)

where *g* is the size of the dependence group, and ⌊*gβ*⌋ is the largest integer smaller than or equal to *gβ*. Note that only *g* − 1 nodes are considered in Eq (2), this is because the chosen node we considered must belong to the giant component (see Eq (1)). For convenience, we rewrite *f*(*R*) in the form of the incomplete beta function,

$$\begin{array}{c} f\left(R\right)=I_{\lambda }\left(a,b\right). \end{array}$$ (3)

Here, *λ* = *p*[1 − *G*_0_(1 − *R*)], *a* = *g* − 1 − ⌊*gβ*⌋ and *b* = ⌊*gβ*⌋ + 1. Thus, Eq (1) can be written as

$$\begin{array}{c} S=p[1-G_{0}\left(1-R\right)]I_{\lambda }\left(a,b\right)=\lambda I_{\lambda }\left(a,b\right). \end{array}$$ (4)

Obviously, when *β* → 1, *I*_*λ*_(*a*, *b*) = 1, Eq (4) reduces to *S* = *p*[1−*G*_0_(1−*R*)], which is just the equation for the classic network percolation [7]. While *β* → 0, *I*_*λ*_(*a*, *b*) = *p* ^*g*−1^[1−*G*_0_(1−*R*)]^*g*−1^, Eq (4) reduces to *S* = *p* ^*g*^[1−*G*_0_(1−*R*)]^*g*^, which is the equation for the percolation on the network with unconditional dependence group [10].

Similarly, we can get the equation for *R*,

$$\begin{array}{c} R=p[1-G_{1}\left(1-R\right)]I_{\lambda }\left(a,b\right), \end{array}$$ (5)

where *G*_1_(*x*) is the generating function of the excess degree distribution *G*_1_(*x*) = ∑_*k*_ *p*_*k*_ *kx* ^*k*−1^/⟨*k*⟩.

Obviously, Eq (5) has a trivial solution *R* = 0, which means that the network has no giant component. With the increasing of *p*, the point that the nontrivial solution of *R* appears for the first time is the critical point (*p*_*c*_, *R*_*c*_). The nontrivial solution of *R* can be presented by the nonzero crossing points of the curves *X*(*R*) = *p*[1−*G*_1_(1−*R*)]*I*_*λ*_(*a*, *b*) and *Y*(*R*) = *R*. Thus, the critical point (*p*_*c*_, *R*_*c*_) corresponds to the tangent of the curves *X*(*R*) and *Y*(*R*), that is

$$\begin{array}{c} p_{c}\left[1-G_{1}\left(1-R_{c}\right)\right]\frac{dI_{\lambda }\left(a,b\right)}{dR_{c}}-p_{c}\frac{dG_{1}\left(1-R_{c}\right)}{dR_{c}}I_{\lambda }\left(a,b\right)=1. \end{array}$$ (6)

Together with Eq (5), we can obtain the critical point of this model.

As we pointed in the last section, our model will reduce to two existing percolation models, whose types of percolation transition are different. So, the crossover of the first and the second phase transitions must can be observed in our model. In the first order transition region, *R*_*c*_ > 0, we can solve Eqs (5) and (6) numerically to obtain the first order transition point $p_{c}^{I}$.

For the second order transition, *R*_*c*_ = 0, thus *λ* = 0. For *a* > 0, Eq (5) is a no solution equation, since *I*_*λ*_(*a*, *b*) = 0. Therefore, in the second order transition region, the parameters must satisfy *a* = 0, i.e., *gβ* ≥ *g* − 1. That is to say the tricritical point *β*_*c*_ is (*g* − 1)/*g*. When *a* = 0 and *λ* = 0, *I*_*λ*_(*a*, *b*) = 1, so Eq (6) gives the critical point of the second order transition,

$$\begin{array}{c} p_{c}^{II}=\frac{1}{G_{1}^{\prime }\left(1\right)},\;\;\;\beta >\beta_{c}. \end{array}$$ (7)

This is the critical point of the classic percolation on a tree-like spare network [7]. This result is obvious as the dependence group does not serve any function when *β* > *β*_*c*_ = (*g* − 1)/*g*. In conclusion, unless the model reduces to the classic percolation model, it always demonstrates a first order phase transition, which is independent of the degree distribution of the network. In other words, the tricritical point of the system only depends on the size of the dependence group.

## ER network

As an example, we consider the cascade process on ER networks, for which the generating function can be written as

$$\begin{array}{c} G_{0}\left(x\right)=G_{1}\left(x\right)=e^{-\langle k\rangle (1-x)}. \end{array}$$ (8)

Substituting these generating functions into Eqs (4) and (5), we get a self-consistent equation for the order parameter *S*,

$$\begin{array}{c} S=p\left(1-e^{-\langle k\rangle S}\right)I_{p(1-e^{-\langle k\rangle S})}\left(a,b\right). \end{array}$$ (9)

Here, the relation *S* = *R* for ER networks is used. Solving this equation, we will obtain the relation of the order parameter *S* and the control parameter *p*.

**Fig 2:**
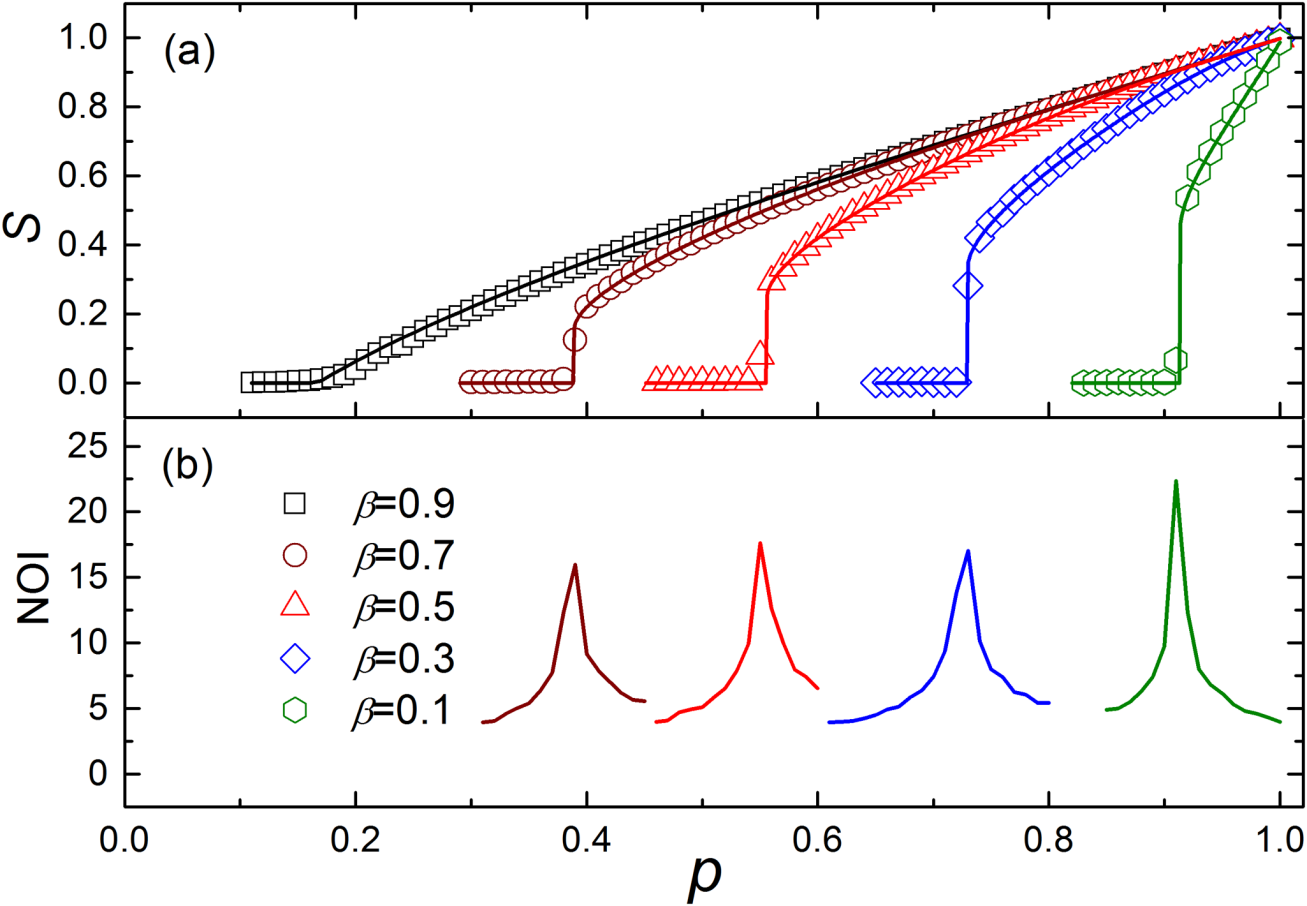
The simulation results of ER networks with 20000 nodes for different *β*. In the simulation, the parameters are set as: ⟨*k*⟩ = 6, *g* = 5. (a) The size of the giant component *S* versus *p*, the fraction of nodes that have been left after random removal. The symbols represent simulation results, and the solid lines show the corresponding analytical predictions of Eq (9). For *β* > (*g* − 1)/*g*, the dependence group has no effect on the cascading process, then the percolation process leads to a second order phase transition. When *β* < (*g* − 1)/*g*, a first order phase transition can be found. Both the first and the second order phase transition processes obey Eq (9). (b) As pointed in ref.[10], the number of iterative failures (NOI) sharply increases when *p* approaches the critical point, so the sharp peaks can identify the corresponding critical points $p_{c}^{I}$ for the first order transition region.

**Fig 3:**
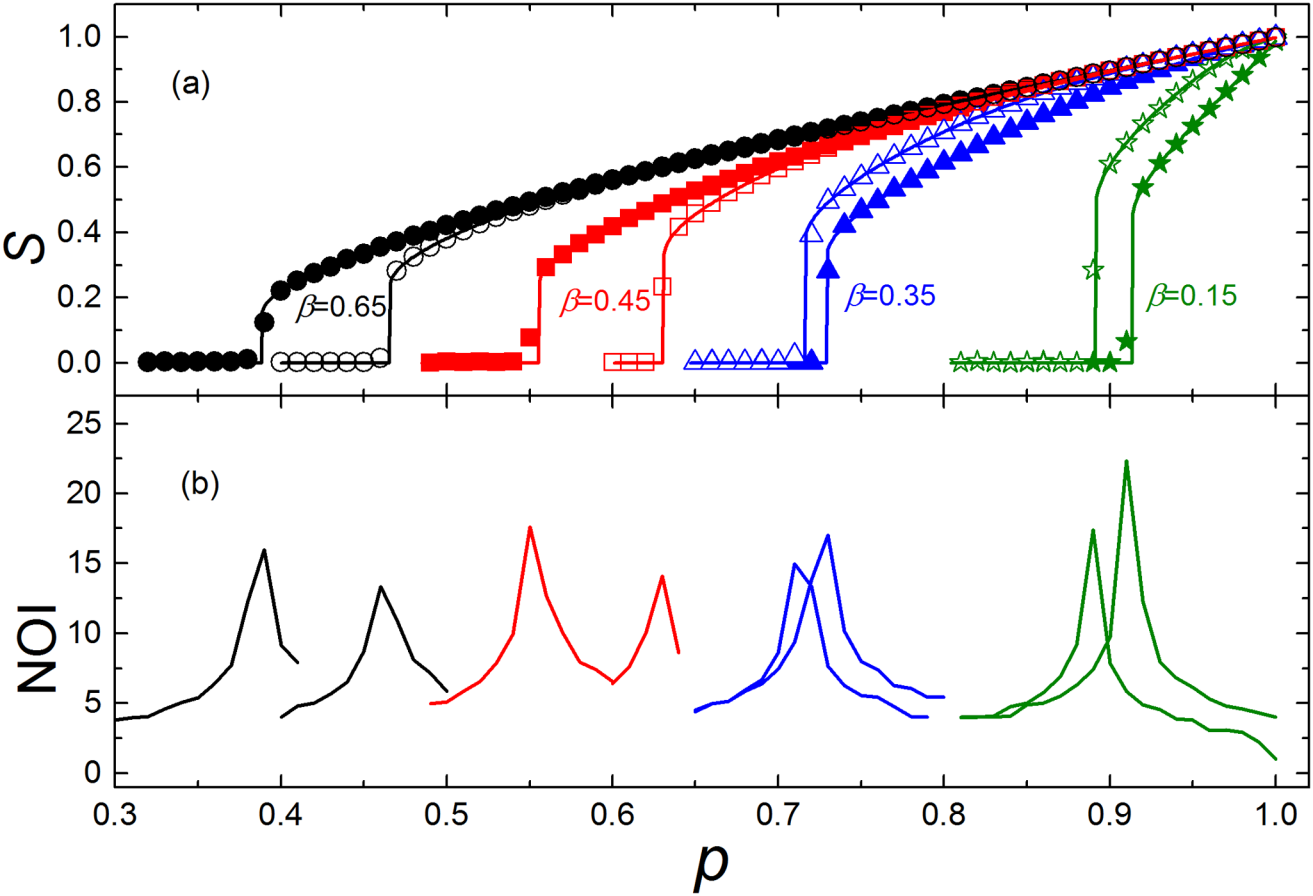
The results of ER networks with 20000 nodes for different *g*. In the simulation, the parameters are set as: ⟨*k*⟩ = 6, *g* = 5 (solid symbols) and *g* = 10 (empty symbols). (a) The size of the giant component *S* versus *p*, the fraction of nodes that have been left after random removal. The symbols represent simulation results, and the solid lines show the corresponding analytical predictions of Eq (9). When *β* = 0.15, 0.35, networks become more robust with *g* increaseing. However, for *β* = 0.45, 0.65, networks become more fragile with *g* increaseing. (b) NOI sharply increases when *p* approaches the critical point, so the sharp peaks can identify the corresponding critical points $p_{c}^{I}$ for the first order transition region.

To validate the theoretical results, we carried out simulations on an ER network with 20000 nodes, and plot the size of the giant component in the end of cascade process as a function of the fraction *p* of nodes that have been left after random removal in Figs 2 and 3. One can see that the simulation results are in agreement with the theoretical results well. From Fig 2, we can find that the network becomes more fragile with *β* decreasing, and when *β* → 1, the system demonstrates a second order phase transition. This is because a small *β* allows the nodes in the same group to have a limited amount of independence.

It is easy to know that a larger dependence group will cause greater damage when it fails. However, for a given *β*, a larger group also has a larger threshold ⌊*gβ*⌋, which means the large dependence groups are robust. These two opposing effects will lead to that the networks with large group sizes are not always more fragile than those with small group sizes (see Fig 3), which is different from the model with unconditional dependence groups [10].

**Fig 4:**
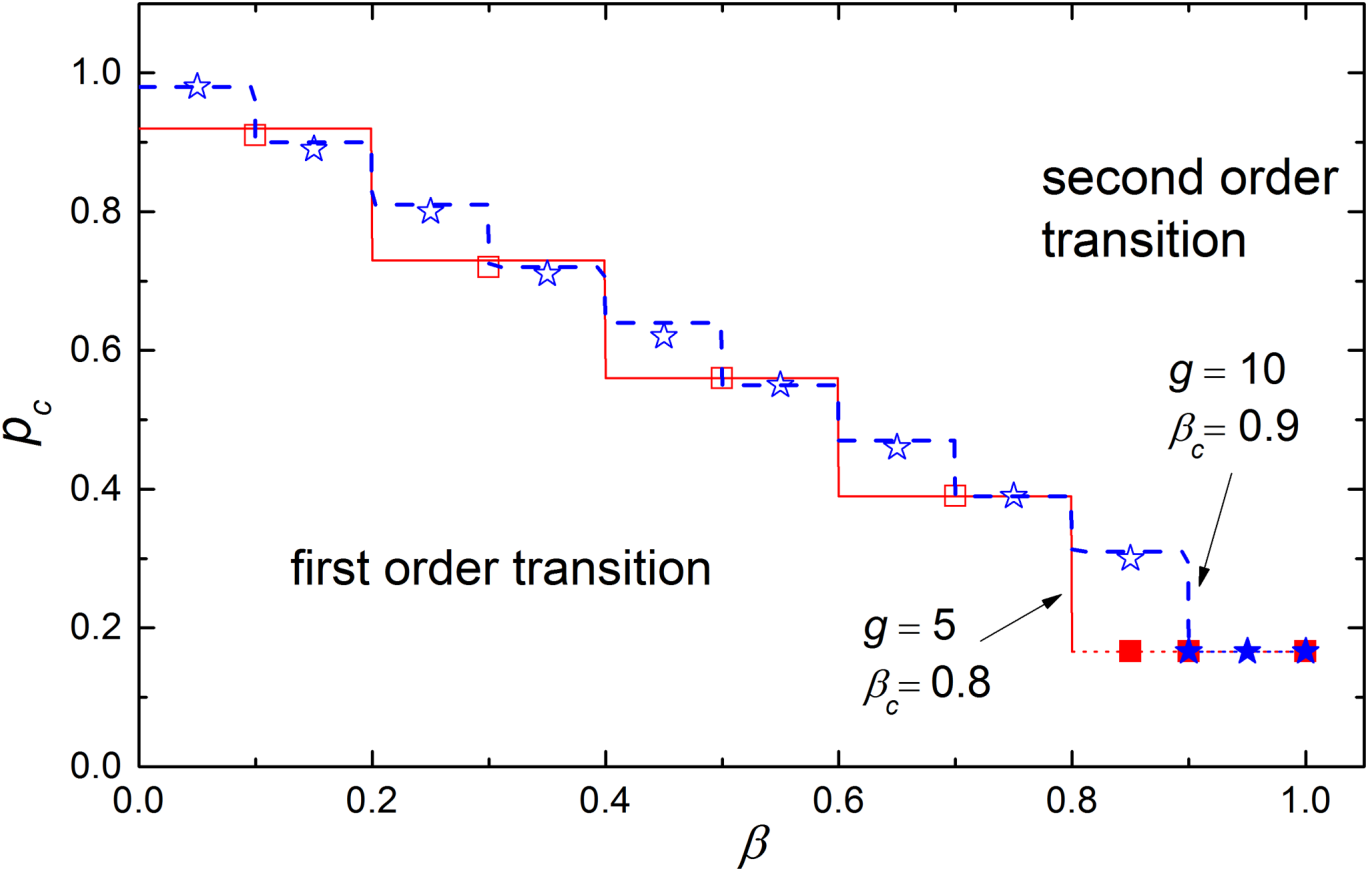
The critical point *p*_*c*_ versus *β*. In the simulation, the network size is set as 20000 and the average degree is ⟨*k*⟩ = 6. The dependence group sizes are set as *g* = 5 (square) and *g* = 10 (star). The solid and dash lines are the corresponding theoretical prediction of Eq (12) for the first order transition region, and the dot lines for the second order transition region. As discussed in the text, the curve of the critical point $p_{c}^{I}$ is discontinuous at *β* = *n*/*g*, *n* = 1, 2, 3, …, *g* −1. For *β* > (*g* − 1)/*g*, the system demonstrates a continuous phase transition. In addition, the critical points of the networks with large dependence group sizes are not always larger than those with small group sizes. This means dependence group size is not the sole factor determining the robustness of such networks.

For given *β*, *g* and *g*′ with *g* < *g*′, the inequality ⌊*gβ*⌋/⌊*g*′ *β*⌋ ≤ *g*/*g*′ must always hold. We find that when ⌊*gβ*⌋/⌊*g*′ *β*⌋ = *g*/*g*′, the network with the larger group size *g*′ is more fragile than the one with group size *g*. While ⌊*gβ*⌋/⌊*g*′ *β*⌋ < *g*/*g*′, the network with the smaller group size is more fragile (see Fig 4).

Next, we will find the first order transition point $p_{c}^{I}$ and the second order transition point $p_{c}^{II}$. For ER networks, we have

$$\begin{array}{c} G_{0}^{\prime }\left(x\right)=G_{1}^{\prime }\left(x\right)=\left\langle k\right\rangle e^{-\langle k\rangle (1-x)}. \end{array}$$ (10)

In addition, the incomplete beta function satisfies

$$\begin{array}{c} \frac{dI_{\lambda }\left(a,b\right)}{d\lambda }=\lambda^{a-1}{\left(1-\lambda \right)}^{b-1}. \end{array}$$ (11)

Substituting these functions into Eq (6), we get a self-consistent equation for the critical point *p*_*c*_,

$$\begin{array}{c} {}[\lambda_{c}^{a}{\left(1-\lambda_{c}\right)}^{b-1}+\frac{S_{c}}{\lambda_{c}}]\frac{d\lambda_{c}}{dS_{c}}=1, \end{array}$$ (12)

where $\lambda_{c}=p_{c}(1-e^{-\langle k\rangle S_{c}})$. Here, we have used Eq (9) and the relation *S* = *R* for ER networks.

For the second order phase transition, *S*_*c*_ → 0, thus $\lambda_{c}^{a}{\left(1-\lambda_{c}\right)}^{b-1}\to 0$, *S*_*c*_/*λ*_*c*_ → 1 and $e^{-\langle k\rangle S_{c}}\to 1$. And then, we will have the critical point for the second order region,

$$\begin{array}{c} \frac{d\lambda_{c}}{dS_{c}}=p_{c}^{II}\left\langle k\right\rangle =1. \end{array}$$ (13)

This is just the critical point for the classic percolation on ER networks, which is consistent with the previous discussion.

For the first order phase transition, *S*_*c*_ > 0, we can not get an analytical expression of the critical point $p_{c}^{I}$. However, we can solve Eqs (9) and (12) numerically in the condition of *S*_*c*_ > 0 to obtain the critical point $p_{c}^{I}$. Because the incomplete beta function is a discontinuous function when *a* and *b* are positive integers, the curve of the critical point $p_{c}^{I}$ versus *β* is discontinuous at *β* = *n*/*g*, *n* = 1, 2, 3, …, *g* −1 as shown in Fig 4.

In Fig 4, we plot the values of *p*_*c*_ as a function of *β* for different *g* and ⟨*k*⟩. As the theoretical analysis, one can find the crossover between the two types of phase transitions, and the tricritical point *β*_*c*_ is only dependent on the size *g* of the dependence group and independent of the average degree ⟨*k*⟩ of the network.

## Discussion and conclusions

Considering the conditional dependence in real networks, we have proposed a cascading failure model on the networks with conditional dependence group. In our model, a dependence group fails only when it has more than a fraction *β* of failed nodes, which covers the model that the failure of only one node can lead to the failure of a dependence group. We think that this will reflect the actual dependence of real networked system in some sense.

Both simulation and analytical results reveal the existence of the crossover between the first and the second order phase transition in our model. When *β* is small, only one or two nodes fail, the dependence group will fail. So the networks can be easily destroyed in the form of the first order phase transition. That is to say, the dependence between nodes is strong. For a larger *β*, the nodes in a dependence group can keep more independence, so the network is more robust. However, the type of percolation is always first order, unless the model reduces to the classic network percolation model. In other words, the tricritical point of our model only depends on the size of the dependence group. Furthermore, we find that larger dependence group size does not always make such networks more fragile, which is meaningful for understanding the robustness of the real networked system with dependence.

## References

[1] Albert R, Barabasi AL. Statistical mechanics of complex networks. Reviews of Modern Physics. 2002; 74: 1–54. 10.1103/RevModPhys.74.47

[2] Dorogovtsev S, Goltsev A, Mendes J. Critical phenomena in complex networks. Reviews of Modern Physics. 2008; 80: 1275–1335. 10.1103/RevModPhys.80.1275

[3] Newman MEJ. Networks: An Introduction. Oxford University Press; 2010.

[4] Albert R, Jeong H, Barabasi AL. Error and attack tolerance of complex networks. Nature. 2000; 406: 378 10.1038/35019019 10935628

[5] Cohen R, Erez K, ben-Avraham D, Havlin S. Resilience of the Internet to random breakdowns. Physical Review Letters. 2000; 85: 4626 10.1103/PhysRevLett.85.4626 11082612

[6] Callaway DS, Newman MEJ, Strogatz SH, Watts DJ. Network robustness and fragility: Percolation on random graphs. Physical Review Letters. 2000; 85: 5468 10.1103/PhysRevLett.85.5468 11136023

[7] Newman MEJ, Strogatz SH, Watts DJ. Random graphs with arbitrary degree distributions and their applications. Physical Review E. 2001; 64: 026118 10.1103/PhysRevE.64.026118 11497662

[8] Bollobás B. Random Graphs. London: Academic; 1985.

[9] Buldyrev SV, Parshani R, Paul G, Stanley HE, Havlin S. Catastrophic cascade of failures in interdependent networks. Nature. 2010; 464: 1025–1028. 10.1038/nature08932 20393559

[10] Parshani R, Buldyrev SV, Havlin S. Critical effect of dependency groups on the function of networks. Proceedings of the National Academy of Sciences of the United States of America. 2011; 108: 1007–1010. 10.1073/pnas.1008404108 21191103 PMC3024657

[11] Bashan A, Parshani R, Havlin S. Percolation in networks composed of connectivity and dependency links. Physical Review E. 2011; 83: 051127 10.1103/PhysRevE.83.051127 21728510

[12] Bashan A, Havlin S. The combined effect of connectivity and dependency links on percolation of networks. Journal of Statistical Physics. 2011; 145: 686–695. 10.1007/s10955-011-0333-5 21728510

[13] Bashan A, Berezin Y, Buldyrev SV, Havlin S. The extreme vulnerability of interdependent spatially embedded networks. Nature Physics. 2013; 9: 667–672. 10.1038/nphys2727

[14] Li M, Liu R-R, Jia C-X, Wang B-H. Critical effects of overlapping of connectivity and dependence links on percolation of networks. New Journal of Physics. 2013; 15: 093013 10.1088/1367-2630/15/9/093013

[15] Li M, Liu R-R, Jia C-X, Wang B-H. Cascading failures on networks with asymmetric dependence. EPL (Europhysics Letters). 2014; 108:56002 10.1209/0295-5075/108/56002

[16] Boccaletti S, Bianconi G, Criado R, del Genio CI, Gómez-Gardeñes J, Romance M, et al The structure and dynamics of multilayer networks. Physics Reports. 2014; 544: 1–122. 10.1016/j.physrep.2014.07.001 PMC7332224 32834429

[17] D’Agostino G, Scala A. Networks of networks: The last frontier of complexity. Berlin: Springer International Publishing; 2014.

[18] Zhou D, Stanley HE, D’Agostino G, Scala A. Assortativity decreases the robustness of interdependent networks. Physical Review E. 2012; 86: 066103 10.1103/PhysRevE.86.066103 23368000

[19] Son S-W, Grassberger P, Paczuski M. Percolation transitions are not slways sharpened by making networks interdependent. Physical Review Letters. 2011; 107: 195702 10.1103/PhysRevLett.107.195702 22181628

[20] Zhou D, Bashan A, Cohen R, Berezin Y, Shnerb N, Havlin S. Simultaneous first- and second-order percolation transitions in interdependent networks. Physical Review E. 2014; 90:012803 10.1103/PhysRevE.90.012803 25122338

[21] Son S-W, Bizhani G, Christensen C, Grassberger P, Paczuski M. Percolation theory on interdependent networks based on epidemic spreading. EPL (Europhysics Letters). 2012; 97: 16006 10.1209/0295-5075/97/16006

